# Owned dog population size and ownership patterns in Costa Rica

**DOI:** 10.3389/fvets.2022.946603

**Published:** 2022-12-01

**Authors:** D. T. Tyler Flockhart, Andrew N. Rowan, John D. Boone

**Affiliations:** ^1^Flockhart Consulting, Saskatoon, SK, Canada; ^2^Wellbeing International, Potomac, MD, United States; ^3^Great Basin Bird Observatory, Reno, NV, United States

**Keywords:** animal welfare, dog ownership, management, free-roaming dogs, companion animal, pet

## Abstract

Dogs (*Canis lupus familiaris*) are one of the most common pets around the world but ownership patterns and human-dog interactions have been changing, particularly in developing nations. We conducted household surveys in Costa Rica to characterize dog ownership, the owned dog population, where dogs were confined at night and in the morning, and behaviors regarding selected dog care issues. We also compared these results to similar questionnaires used in Costa Rica over the past 20 years. We found 76% of households in Costa Rica owned at least one dog and on average there were about 1.4 dogs owned per household. These dog ownership rates are higher than previous estimates. The probability of owning a dog was highest on farms and lowest in single family dwellings without a yard, higher among respondents that owned their homes and decreasing with increasing human population density The total number of owned dogs in Costa Rica was estimated to be 2,222,032 (95% confidence intervals: 1,981,497–2,503,751). The sterilization rate for homed dogs in 2020 was approximately 62% (females: 67%, males: 61%) which is higher than the 18% of owned dogs that were sterilized in a 2003 survey. Overall, only 1.2% (95% CI: 0.3–2.5%) of owned dogs slept on the street with a slightly higher proportion on the street at 8 am. The number of owned dogs roaming the streets at night nation-wide was estimated to be 27,208 (95% CI: 7,557–56,619) compared to 43,142 (95% CI: 20,118–73,618) on the street at 8 am. The number of unowned free-roaming dogs in Costa Rica has never been estimated but we can generate some idea of the size of the unowned dog population by determining the proportion of free-roaming dogs on the street wearing collars. There was a negative relationship between human population density and owned dogs being on the street meaning fewer dogs roam the streets in highly populated areas compared to less populated areas. Overall, we identify trends against which future progress can be measured and provide information that are critical in designing effective humane dog management programs in Costa Rica in the future.

## Introduction

Dogs (*Canis lupus familiaris*) are one of the most common pets around the world and play important roles in owners lives including companionship and protection (1). Free-roaming dogs, comprised of owned dogs that have unconstrained outdoor access some or all of the time and unowned dogs that move freely outside, can be vectors of disease (2), have impacts on the environment (3), and can cause injuries from interactions with people (4). In developing nations, especially those in tropical areas, dog ownership and outdoor access may be different than in fully developed nations (5). Further, the tolerance of free-roaming dogs may vary over space and time and depend on local context (6, 7). Permissive attitudes regarding free-roaming dogs in some countries, coupled with the perceived efficacy of sterilization as a dog management tool (8) could result in large number of free-roaming dogs. In turn, a large population of free-roaming dogs may lead to liability problems and risks to humans from interactions with dogs (9). Dog bites to humans are a risk wherever there are free-roaming dogs (10). Dog bite incidence may decrease following dog sterilization (11) presumably because agonistic interactions among dogs competing over a bitch in heat and from female dogs protecting puppies should be absent among sterilized dogs (12). Developing suitable management plans (e.g., vaccinating a certain proportion of free-roaming dogs per month) to reach societal objectives (e.g., reducing rabies prevalence in free-roaming dogs by 80%) requires, at a bare minimum, reasonable estimates of dog ownership, dog outdoor access, and population size but few estimates of these basic metrics are available (13).

Human-dog interactions have been changing around the world over the past fifty or more years. In North America in 1950, 30% of pet dogs were considered to be owned free-roaming dogs (14) yet today most pet dogs in North America are closely controlled (*via* leashes and visible and invisible fences) and owned free-roaming dogs probably constitute less than 1% of the total US dog population (unpublished data, ANR). Something similar to the changes that occurred in the USA in the last half of the 20^th^ century appear to be happening now in Costa Rica. Freely roaming dogs were common in and around Heredia, Costa Rica in the 20^th^ century but have declined significantly in the past 20 years [from observations by one of us (ANR) during visits to Costa Rica in 1999 and again in 2015]. We speculate that the presence of active shelter and education programs in the Heredia region (and in the San Jose valley) has led to changes in human-dog interactions in the San Jose valley but have no direct causal data supporting such a link.

This study describes survey work conducted in Costa Rica to generate a current baseline of dog ownership patterns, derive a population estimate across the country, and compare these results to previous surveys focused on dog ownership. The current surveys aimed to document continuing changes in dog ownership behaviors in Costa Rica based on a randomized, nationwide household survey. Two prior surveys of dog ownership in Costa Rica found that approximately half of all households owned a dog and that these homes owned on average about 1.6 dogs. These previous results provide a rare opportunity to compare the current study to previous conditions to monitor changes in the human-dog interaction over time.

## Methods

### Survey design and methods

We used a household survey, statistical modeling, and qualitative comparisons to understand patterns of dog ownership, outdoor access, and population size across Costa Rica. The survey was administered between August and September 2020 by TGM (https://tgmresearch.com/; Ho Chi Minh City, Vietnam) and posed questions to households about pets and animal-related issues *via* the internet or cell phones. Respondents were selected from registered online and mobile panels of participants from Costa Rica and restricted to adults only. The household survey focused on “owned” dogs regardless of their confinement status and recorded the number of owned dogs, where dogs sleep at night, and where dogs are at 8 am. We asked about where dogs were at 8 am to better understand the proportion of owned dogs that may be outside and unconfined at a time when dogs might be expected to be let outside after spending the night indoors or confined. We also asked if owned dogs were vaccinated against rabies, if they were sterilized, or had visited a veterinarian in the past year. For dogs that had visited a veterinarian in the past year we asked if the dog had visited for regular routine care or emergency care which provides some insight as to owner provisions of preventative care vs. emergency interventions. The survey recorded geolocation data so that dog ownership and human density of each household could be analyzed. The questionnaire used for the Household Survey is provided in Appendix 2.

Approximately half the human population of Costa Rica lives in the central valley around the capital, San Jose, but we were interested in determining country-level estimates that represented the national demographics. The country is divided into seven provinces that are further subdivided into 82 cantons and 488 districts. To obtain a representative sample while recognizing sample size and logistical constraints, we geographically stratified our sampling to match the proportion of the population in each province (Table 1) but oversampled in the Province of Heredia because we wanted to determine the extent to which residents in Heredia knew of and made use of a particular local shelter–the Asociacion Humanitaria.Para Proteccion Animal de Costa Rica (AHPPA), a partner in our ongoing research.

**Table 1:** Basic descriptive statistics for each province of Costa Rica and nationally of various attributes of dog ownership and interactions from a national household survey conducted in September 2020.

**Province**	**Respondents**	**% Human population**	**# Dogs per household**	**% with dogs**	**Persons /HH**	**% Homes owned**	**Ave. # roaming dogs fed by neighbors**	**% Households with dog bite in last year**
Alajuela	215 (16%)	20%	1.55	81.9%	3.71	65.6%	6.7	17.5%
Cartago	164 (12%)	11%	1.38	78.0%	4.05	68.3%	2.6	17.1%
Guanacaste	64 (5%)	8%	1.31	78.1%	4.33	40.6%	3.1	17.2%
Heredia	255 (19%)	10%	1.34	71.8%	3.75	51.4%	1.8	14.7%
Limon	66 (5%)	9%	1.77	87.9%	3.85	59.1%	2.2	12.3%
Puntarenas	96 (7%)	10%	1.51	81.3%	4.02	56.2%	5.8	14.9%
San Jose	489 (36%)	33%	1.38	73.4%	3.98	56.4%	1.9	16.2%
Total	1,349 (100%)	100%	1.42	76.5%	3.92	57.7%	3.1	16.0%

Before analysis, we used criteria to remove incomplete or biased responses. The criteria for removing responses were included the following:

a) if the geographic location was outside Costa Rica,
b) if the geographic location had identical latitude/longitude values with another location (when more than one person from the same household responded),
c) if the number of people in the household was >50 or zero,
d) if the dwelling type was not a farm, multi-unit building or single family home,
e) if the dwelling ownership was not either owned or rented;
f) or when the location was outside the spatial data sets (i.e., land use and population density) used to predict total dog population.

The final data set included 1,349 responses across the country from an initial sample of 1,626 responses.

### Data analysis

Four analyses were conducted to understand dog ownership, the dog population, and dog confinement situation. A binomial generalized linear model (logistic regression) was used to explain the probability of dog ownership. A Poisson generalized linear model was used to explain the number of dogs owned per household. Multinomial generalized linear models were used to explain where dogs slept at night and where owned dogs were at 8 am. The explanatory variables that were considered in each model included:

a) age of the respondent (in both linear and quadratic forms),
b) dwelling type (farm, multi-unit building, single family home with yard, single family home without yard),
c) household size, and
d) dwelling ownership status (own or rent).

Additionally, geographic variables were extracted from the location of the respondents' residence including

a) elevation (considered in both linear and quadratic forms),
b) land use (5 categories:
  i) Broadleaf evergreen forest = 1,000 respondents,
  ii) Cultivated and managed terrestrial area = 288 respondents,
  iii) Built up areas = 30 respondents,
  iv) Broadleaf closed to open = 17 respondents,
  v) Shoreline = 11 respondents; (15),
c) province, canton, and population density at 1 km resolution (16).

The variable of elevation was used as a predictor for two reasons. First, given the large altitudinal relief differences found in Costa Rica (sea level to 3821 m) we were cognizant of the possibility of higher elevations having extreme conditions that might limit dog distributions at high elevations. In this case, we would have predicted lower dog abundance at higher elevations in a linear fashion. Second, given the observation that 70% of the population lives in the central valley, we wanted to account for the general observation that pet ownership per household is generally reduced in highly urbanized areas relative to rural areas (17). In this case, we would predict a curved relationship with the lowest dog abundance occurring at the elevation corresponding with the large, dense human population found in the central valley. Elevation could therefore be a useful metric for modeling spatial patterns across large geographies while simultaneously explaining variation in dog ownership patterns caused by environmental constraints and dense human populations that correspond with geographic anomalies.

All analyses were conducted using a stepwise model selection procedure to determine the most parsimonious explanatory model. The process began with a null model (intercept-only) and then moved on to models with singular effects of the variables listed above. Individual variables were added in a stepwise manner to check for improved model fit. Models that did not show a reduction in AIC (Akaike Information Criterion–see below) were ignored whereas models with improved fit were carried forward with the addition of further variables (18).

The models were ranked using AIC corrected for small sample size (AICc) where the model with the lowest AICc value is considered the best fit of the data. Models within 2 AICc units of the top model (<2 ΔAICc) are considered competing models and hold some support to explain the data (18). In cases where the competing models are simpler, or nested, versions of the top model, the added parameters of the top model are unlikely to provide much explanatory power and, in these cases, the simpler model is considered the most parsimonious (19). For each analysis, we present the mean and 95% confidence interval parameter estimates of the most parsimonious model.

To derive a country-level estimate of dog population size required extrapolating model estimates and associated uncertainty across the range of demographic and geographic variation in Costa Rica. Bootstrapping was therefore used to estimate dog population abundance across the diverse conditions found in Costa Rica and the proportion of dogs confined. Because our data was derived at the household-level and models contained geographic explanatory variables (e.g., elevation) extracted from the respondents location, the bootstrapping procedure required a location of an average random household during each simulation but we did not have a shapefile with household locations at any scale. To overcome this obstacle, we randomly placed 10,000 points within each of the 81 Cantons weighted by population density. Using this approach, the random locations were predominately in areas with the highest housing density and corresponding elevation. This approach accounted for variation in these variables on the landscape but appropriately weighted those factors based on clustering of households in urban areas. The point locations were used to extract both human population density, elevation, and land use values for the bootstrap simulations. As an example of the random point generation, Supplementary Figure S1 shows the population density and elevation for one Canton and the 10,000 random locations weighted by population density.

Each bootstrap simulation resampled the datasets by randomly selecting 2/3 of the original dataset with replacement. We then refit the best supported regression models from the model selection procedure described above and used the parameter estimates to derive predictions for each Canton. The predictions for each Canton were mean number of dogs per household. This figure was then multiplied by the number of households in the Canton to derive an estimate of the total number of owned dogs in the Canton. We also predicted the proportion of dogs in a Canton that were confined at both 8 am and at night (asleep in the house), on the street or in the yard. By multiplying the predicted population size in a Canton by these proportions we could estimate the number of dogs in each Canton confined in the house, on the street, or in the yard. By summing the population estimates of each Canton we produced an estimate of the number of owned dogs in Costa Rica. We then calculated the mean and 95% confidence intervals of the bootstrapped estimates to derive national population statistics. All modeling was conducted in Program R version 4.1.2 (20).

### Comparison to previous population estimates

Our qualitative assessment of changes in the human-dog relationship was done by comparing our population estimates to previously conducted surveys in Costa Rica. Two surveys were carried out by the World Society for Protection of Animals (WSPA–now World Animal Protection) in 2003 and 2011 but were focused on respondents living in the San Jose metro area. A national survey of the ownership of pet wildlife (but including pet dogs and cats) was also undertaken in 2000 asking if a household owned dogs but did not quantify the number of dogs per household (21). The study by Drews (21) surveyed 1,021 households based on random selection of 278 of the total 10,535 census segments across the country. Households were randomly selected for surveys until the sex and age quota for that segment was covered. Our survey (2020) incorporates questions that were asked in previous surveys and therefore, there is reasonably good “pre-treatment” data, which ultimately facilitates understanding changes in dog ownership patterns over the past two decades. We compared dog ownership patterns, number of dogs per household, national estimated dog population size, confinement location of dogs at night, and whether dogs were vaccinated against rabies, had been sterilized, or had visited a veterinarian during the past year. Given the similar methodology among the surveys (21), the comparison provides the opportunity to understand broad changes in dog ownership and help design a strong monitoring program as an integral component of the ongoing management program, rather than addressing monitoring as an afterthought.

## Results

**Figure 1:**
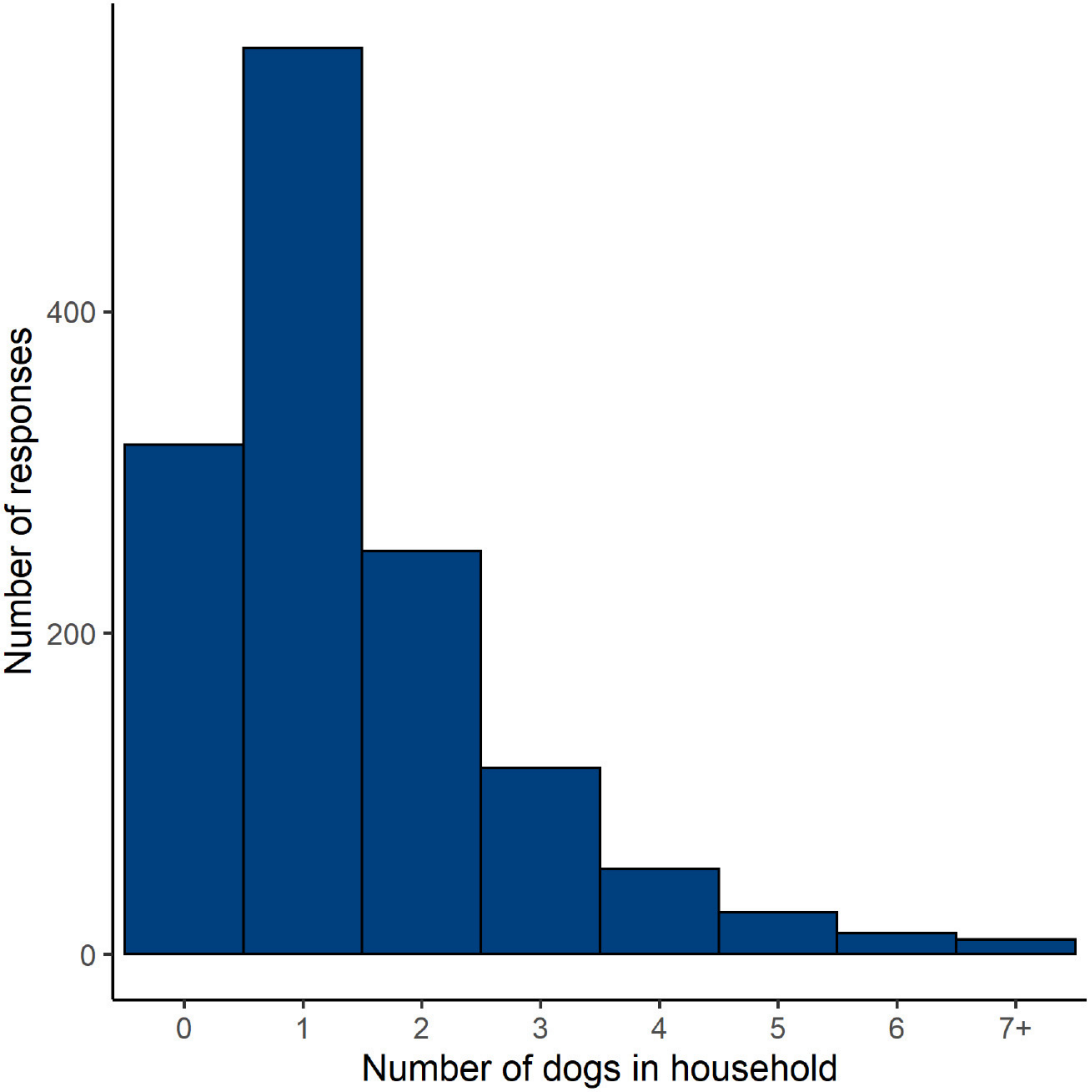
Histogram of the number of owned dogs per household from a survey of 1,349 respondents in Costa Rica.

According to our survey results, 76% of households in Costa Rica own at least one dog (Figure 1) and on average there were 1.4 dogs owned per household (Table 1). The estimated mean number of roaming dogs fed by the neighbors of survey respondents ranged from 1.8 in Heredia Province to 6.7 in Alajuela Province and was 3.1 dogs across the country (Table 1). The proportion of households where one or more people were bitten by dogs in the past year was 16% and did not vary much by province (Table 1). The majority of respondents owned their home except in Guanacaste.

**Figure 2:**
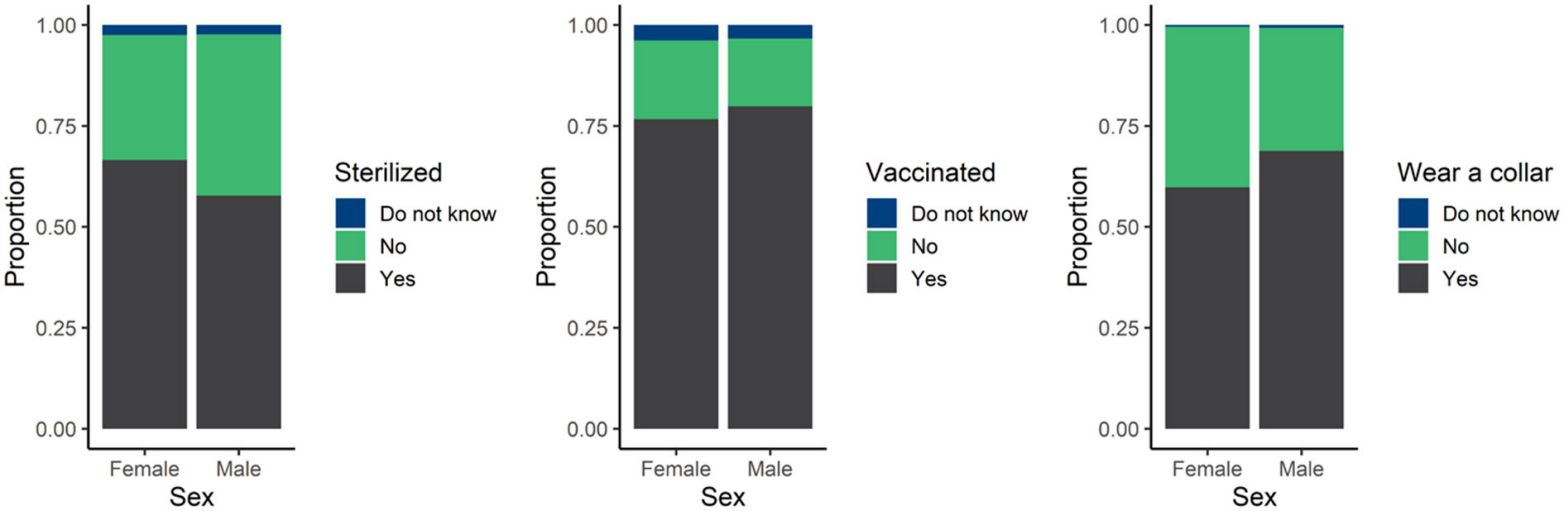
Proportion of male and female dogs that were sterilized, vaccinated, and wore a collar.

**Figure 3:**
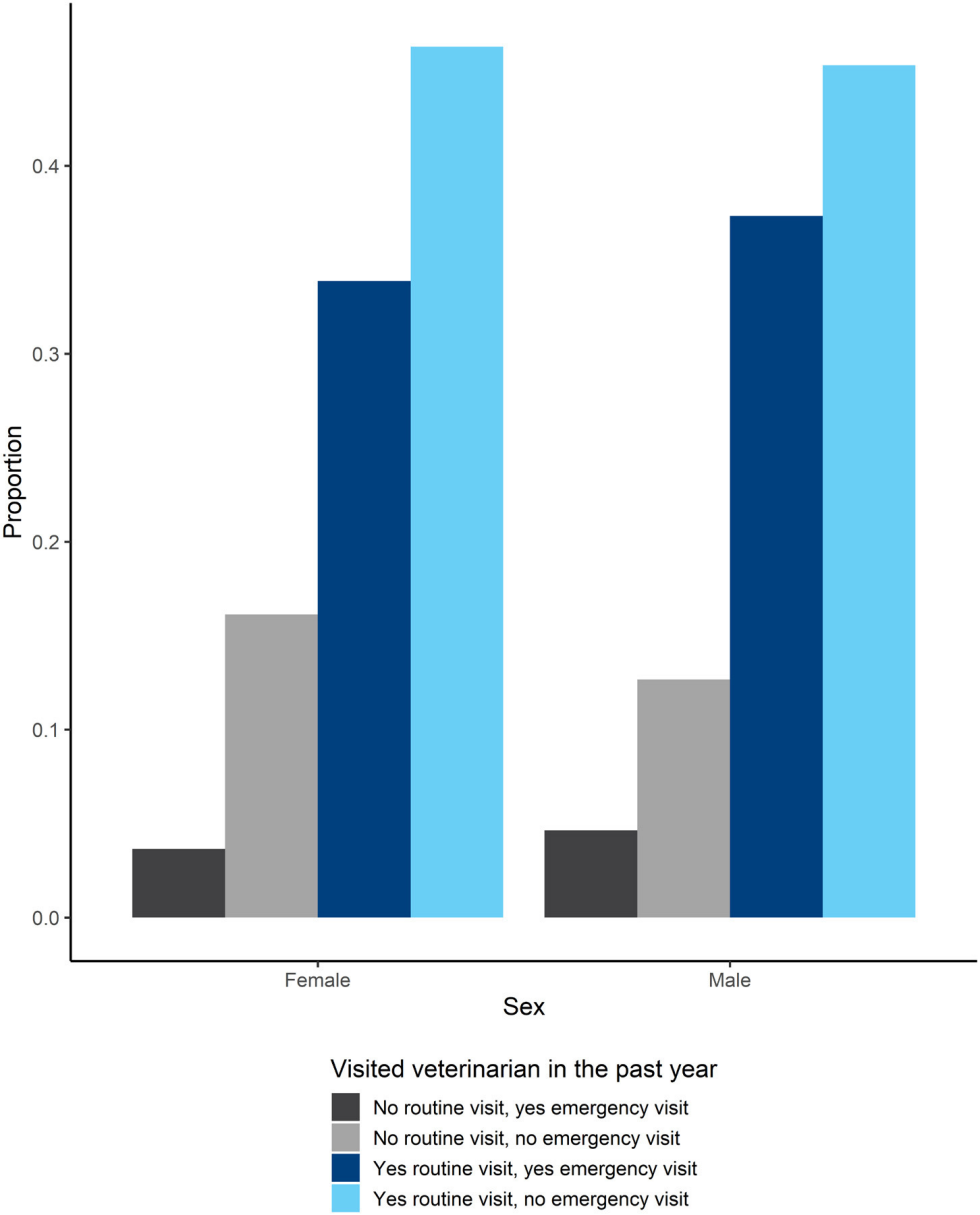
Proportion of male and female dogs whose owners knew if they had visited a veterinarian in the past year for routine care and emergency care.

The majority of owned dogs were sterilized (61%), vaccinated (79%), and wore a collar (65%; Figure 2). Males were more likely to be vaccinated and wear a collar compared to females but less likely to be sterilized. More than three quarters of owned dogs had visited a veterinarian for routine care in the past year and about 35% of those dogs visited a veterinarian for emergency care. Of dogs that did not visit a veterinarian for routine care in the past year, only about 15% of those dogs visited a veterinarian for emergency care. There was a slight preponderance of males visiting a veterinarian for both routine and emergency care compared to females (Figure 3).

The best model to explain dog ownership included dwelling type, dwelling ownership, population density and a quadratic form of elevation (Table 2). The probability of owning a dog was highest on farms (92%) and lowest in single family dwellings without a yard (56%), higher among respondents that owned their homes (66%) compared to rented (50%), lowest at high human population density, and lowest at mid elevations around 800 m (Supplementary Table S1). The full list of candidate models is provided in Supplementary Table S2.

**Table 2:** A reduced candidate model list ranked using Akaike Information Criterion (AIC) to explain the probability of dog ownership of residents of Costa Rica.

**Model**	**K**	**AICc**	**ΔAICc**	**Li**	**Wi**	**LL**	**∑Wt**
Dwelling type + Ownership + Population density + Elevation^2^	9	1,367.1	0	1	0.34	−674.48	0.34
Dwelling type + Ownership + Population density + Elevation^2^ + Land use	15	1,367.6	0.5	0.78	0.27	−668.62	0.61
Dwelling type + Ownership + Population density + Elevation^2^ + Household size	10	1,368.32	1.22	0.54	0.19	−674.08	0.8
Dwelling type + Ownership + Population density + Elevation^2^ + Household size + Land use	16	1,368.84	1.74	0.42	0.14	−668.21	0.94
Dwelling type + Ownership	6	1,370.62	3.52	0.17	0.06	−679.28	1

The model producing the lowest AICc value is the best one. Variables considered include age [linear (Age) or quadratic (Age^2^) form], land use, dwelling type, dwelling ownership status, population density, elevation (in linear or quadradic forms), household size, Province, and Canton. The Table includes the following columns: K–number of parameters, AICc–correction for small sample size, ΔAICc–difference between AICc for each model relative to the top model, Li–model likelihood, Wi–Akaike Information Criterion model weight, LL–model Log-likelihood, and ∑Wt–cumulative Akaike weight.

**Figure 4:**
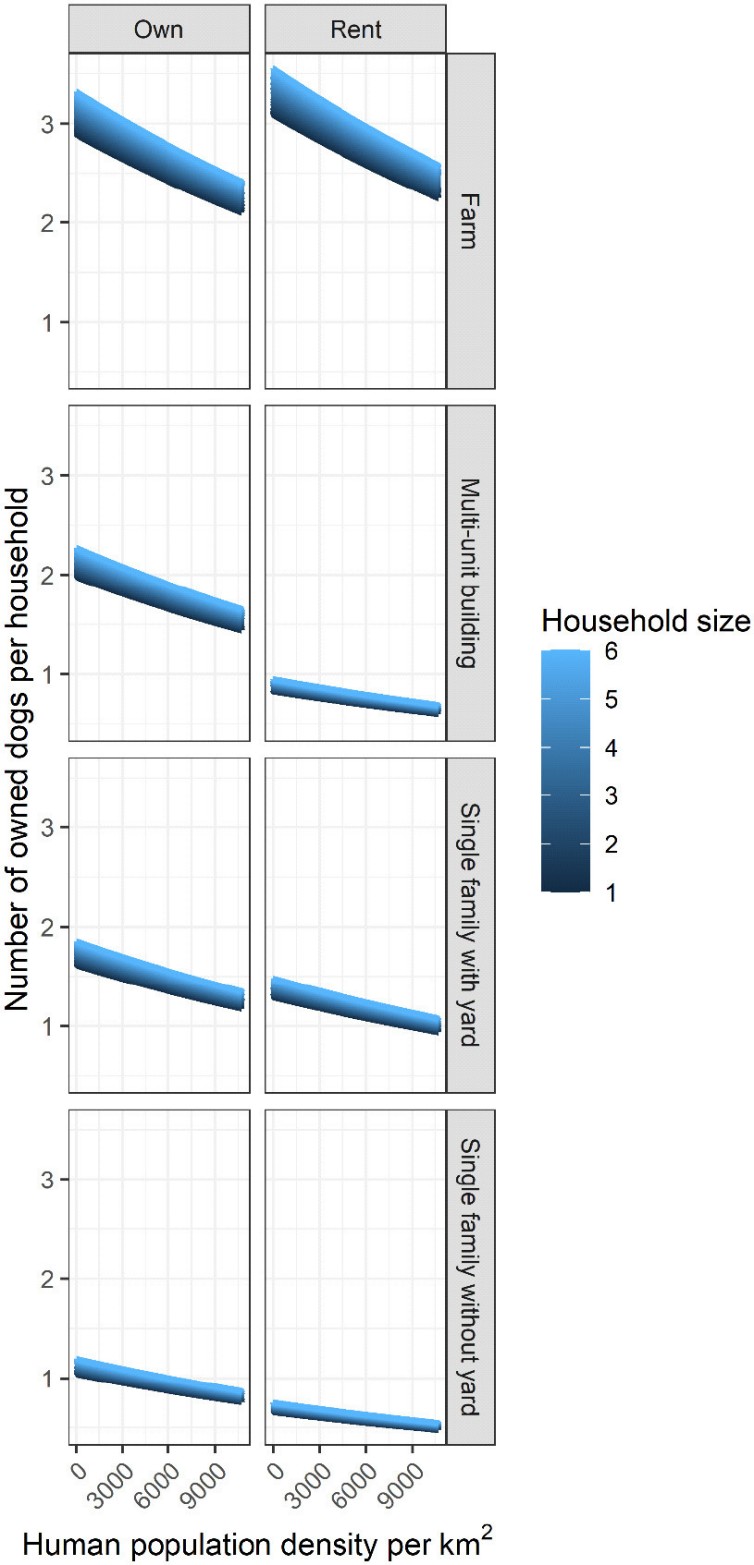
Mean predicted number of owned dogs by population density, household size, dwelling ownership type, and ownership status among residents of Costa Rica. All predictions are standardized to the mean elevation of 961 m for all respondents.

The number of owned dogs per household was best explained by dwelling type and ownership status, elevation, population density, and household size (Table 3). Age, Province, and Canton did not appear to strongly influence the number of owned dogs per household (Supplementary Table S3; although see Supplementary Tables S4, S5 for these estimates). Larger households had more owned dogs while more dogs per household occurred at low human population density (Figure 4). There was an interaction between dwelling type and ownership (Table 4) whereby the highest number of dogs per household occurred on rented farms while the lowest occurred in rented single family homes without yards (Figure 4). The total number of owned dogs in Costa Rica was estimated to be 2,222,032 (95% confidence intervals: 1,981,497–2,503,751).

**Table 3:** Reduced candidate model list of models < 10 ΔAICc ranked using Akaike Information Criterion (AIC) to explain the number of dogs owned per household of residents of Costa Rica.

**Model**	**K**	**AICc**	**ΔAICc**	**Li**	**Wi**	**LL**	**∑Wt**
Dwelling type * Ownership + Population density + Elevation^2^+ Household size	16	4,120.93	0	1	0.81	−2,044.26	0.81
Dwelling type * Ownership + Population density + Elevation^2^	15	4,125.32	4.38	0.11	0.09	−2,047.48	0.9
Dwelling type + Ownership + Population density + Elevation^2^+ Household size	10	4,125.54	4.6	0.1	0.08	−2,052.69	0.98
Dwelling type + Ownership + Population density + Elevation^2^	9	4,129.52	8.58	0.01	0.01	−2,055.69	0.99
Dwelling type + Ownership + Population density + Elevation^2^ + Household size + Land use	16	4,130.59	9.65	0.01	0.01	−2,049.09	1

Variables in the models include age in either a linear (Age) or quadratic (Age^2^) form, land use, dwelling type, dwelling ownership status, population density, elevation (in linear or quadradic forms), household size, Province, and Canton. For each variable are the Akaike Information Criterion corrected for small sample size (AICc), difference between AICc for each model relative to the top model (ΔAICc), model likelihood (Li), Akaike Information Criterion model weight (Wi), cumulative Akaike weight (∑Wt), number of parameters (K) and model Log-likelihood (LL).

**Table 4:** Parameter estimates and 95% confidence interval of the top model to explain the number of dogs per household owned by residence of Costa Rica.

**Parameter**	**Estimate**	**95% confidence interval**
Intercept	0.596	−0.033–1.226
Dwelling type:		
Multi-unit building	−0.122	−1.042–0.798
Single family with yard	−0.106	−0.729–0.517
Single family without yard	−0.906	−1.714–−0.097
Dwelling ownership:		
Own	0.433	−0.229–1.094
Rent	0.499	−0.280–1.278
Population density	−2.981e-5	−5.027e-5–−9.344e-6
Elevation	−5.315e-5	−3.521e-4–2.458e-4
Elevation^2^	6.001e-8	−1.055e-7–2.255e-7
Household size	0.028	0.007–0.049
Multi-unit building: Own	−0.257	−1.257–0.743
Single family with yard: Own	−0.476	−1.161–0.209
Single family without yard: Own	−0.116	−1.005–0.773
Multi-unit building: Rent	−1.197	−2.267–−0.127
Single family with yard: Rent	−0.769	−1.573–0.035
Single family without yard: Rent	−0.647	−1.630–0.336

Intercept represents a farm.

**Figure 5:**
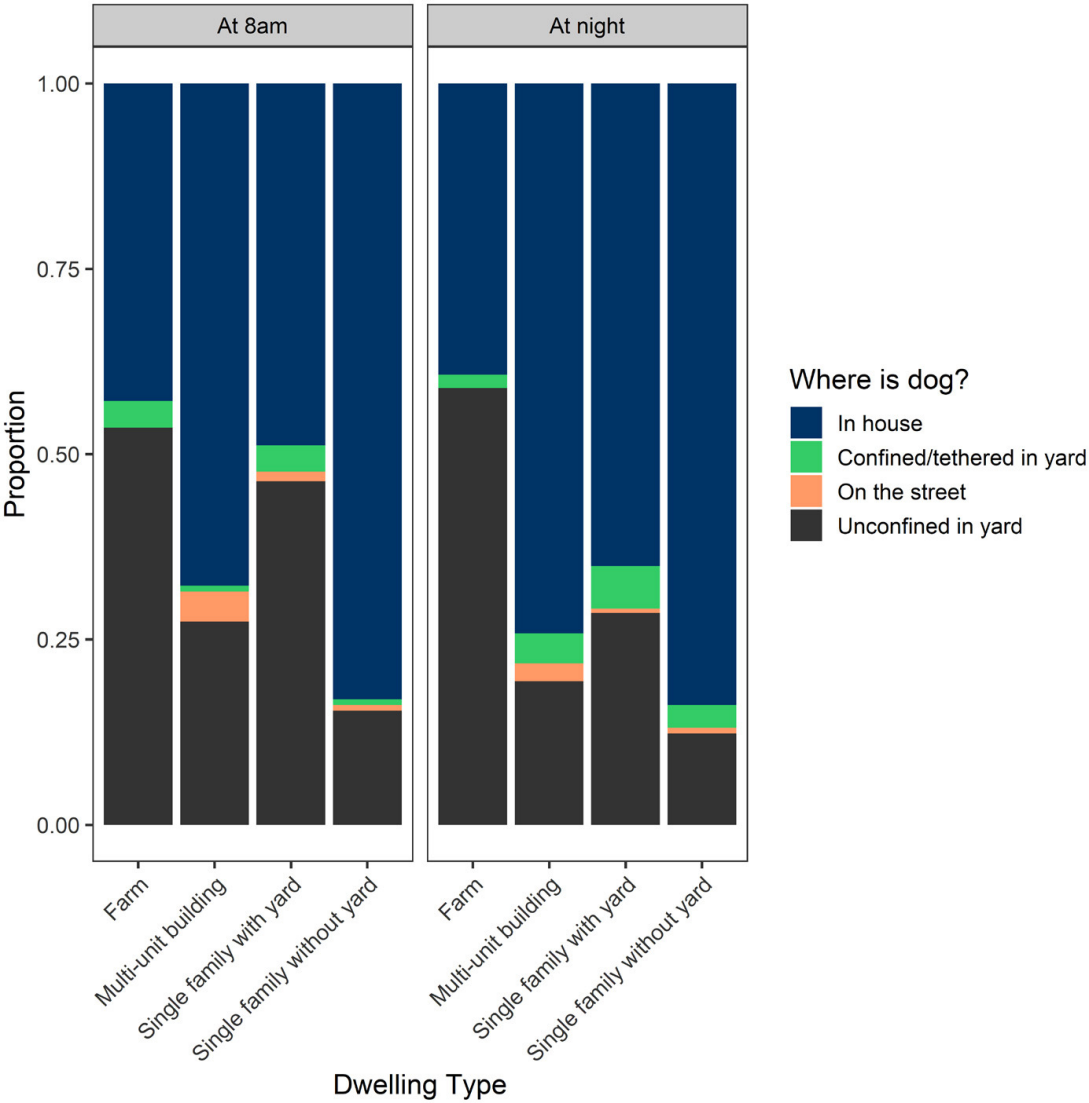
Descriptive statistics of where the proportion of owned dogs in Costa Rica occur at night and at 8 am by dwelling type as reported by dog owners.

The best predictors of where a dog was confined at night and at 8 am were elevation, dwelling type and human population density (Table 5). There was little effect of dog owners' age, province or canton of residence, land use, household size, dwelling ownership status, or land use on where dogs were confined (Supplementary Tables S6, S8). The majority of dogs were confined in the house or unconfined in the yard at night (in house: 66%; unconfined in yard: 28%) and in the morning (in house: 53%; unconfined in yard: 43%; Figure 5). Overall, only 1.2% (95% CI: 0.3–2.5%) of dogs slept on the street with a slightly higher proportion on the street at 8 am (Figure 5). There was a negative relationship between human population density and dogs being on the street or in the yard (both tethered and free) at night and at 8 am indicating that more dogs are confined to the house in more heavily populated areas (Supplementary Tables S7, S9). Therefore, there would likely be fewer dogs roaming on the street in highly populated areas compared to less populated areas. The number of owned dogs roaming the streets at night nation-wide was estimated to be 27,208 (95% CI: 7,557–56,619) compared to 43,142 (95% CI: 20,118–73,618) on the street at 8 am (amounting to only 1–2% of owned dogs in the country.

**Table 5:** Reduced candidate model list of models <10 ΔAICc ranked using Akaike Information Criterion (AIC) to explain where owned dogs were confined at night and at 8 am according to dogs owners of Costa Rica.

**Model**	**K**	**AICc**	**ΔAICc**	**Li**	**Wi**	**LL**	**∑Wt**
*At night*							
Elevation^2^ + Population density + Dwelling type	21	2,729.94	0	1	0.59	−1,343.7	0.59
Elevation^2^ + Dwelling type	18	2,731.59	1.65	0.44	0.26	−1,347.6	0.85
Elevation^2^ + Population density + Dwelling type + Province	39	2,733.34	3.4	0.18	0.11	−1326.74	0.96
Elevation^2^ + Dwelling type + Province	36	2,735.21	5.26	0.07	0.04	−1,330.81	1
*At 8 am*							
Elevation^2^ + Population density + Dwelling type	21	2,868.00	0	1	0.98	−1,412.72	0.98
Elevation^2^ + Population density + Dwelling type + Province	39	2,876.35	8.35	0.02	0.02	−1,398.24	1

For each variable are the Akaike Information Criterion corrected for small sample size (AICc), difference between AICc for each model relative to the top model (ΔAICc), model likelihood (Li), Akaike Information Criterion model weight (Wi), cumulative Akaike weight (∑Wt), number of parameters (K) and model Log-likelihood (LL).

### Comparison to previous owned dog population estimates

There were three previous surveys of the owned dog population in Costa Rica; the results of the four surveys are provided in Table 6. In 2001, Carlos Drews reported that 53% of Costa Rican households had one or more dogs. In 2003 and 2011, the World Society for the Protection of Animals surveyed dog ownership in the San Jose metro area and reported on the dog ownership rates as well as on other human-dog relationships.

**Table 6:** Four dog surveys in Costa Rica in present century.

**Statistic**	**Drews (National)**	**WSPA** ** (San Jose Metro)**	**WSPA (San Jose Metro)**	**This study** ** (National)**
Year	2000	2003	2011	2020
% Homes with dogs	53%	50%	49%	76.5%
Dogs per owning household		1.65	1.67	1.86
Human population	3,962,372	4,164,053	4,633,086	5,094,118
Number households	1,100,000	1,190,000	1,300,000	1,509,789
Estimated dog population	970,000	981,800	1,063,800	2,222,030
Dogs per 1,000	245	236	230	436
Daytime–dog in house		19.5%	49.5%	
Nighttime–dog in house		26.5%	54.2%	65.9%
Vaccinated against rabies			75.5%	78.5%
At least one sterilized		18%	36%	61.5%
Visit the vet during year		61%	80%	76.5%

The current (2020) survey identified a much higher percentage of households owning dogs (76% vs. around half in the three previous surveys) and a higher number of dogs (1.86) per owning household. According to the latest survey, the number of dogs in Costa Rican households is double the estimate from about 10 years earlier (increased from around 1.06 million to 2.22 million).

However, there are signs from other questions in the survey that the interaction with dogs of the average Costa Rican household has changed quite dramatically. In 2003, only 27% of dogs slept indoors at night and <20% were sterilized. In 2020, two-thirds of dogs were sleeping indoors at night and almost two-thirds were sterilized. These results indicate a substantial change in dog care among Costa Rican households in this century.

On average, the WSPA surveys reported that around 50% of Costa Rican households “own” dogs and that each owning household had an average of 1.67 dogs (for a total estimated dog population in 2011 in the metro area of 583,000).

The two WSPA surveys also documented substantial changes in human behavior over the 8 years from 2003 to 2011 (Table 6). These changes reflect an increase in “responsible” pet owner behavior with the average age of the dogs increasing and twice as many dogs sleeping indoors at night in 2011 compared to 2003. There was also an increase in the proportion of dogs being taken to a veterinarian every year and a halving of the number of dogs being allowed to roam outside without supervision. The survey this year (2020) indicates that the human-dog relationship continued to strengthen since 2011 (higher dog sterilization rates and higher proportion of owned dogs sleeping indoors).

## Discussion

We produced models to explain dog ownership, the dog population, and locations where dogs are at different times of the day in Costa Rica based on a representative sample of residents. Our results suggest that the number of households that owned dog numbers may have increased (although these changes may be an artifact of the survey methods). Most dogs are confined to the house or yard overnight and early in the morning which amounts to few owned dogs roaming on the street during these times. Additionally, over the past two decades, the proportion of dogs that spend the night in the house has more than doubled. The vast majority of dogs in our survey had visited a veterinarian for routine care over the past year, were vaccinated, and had been sterilized. The patterns observed suggest a shift in care conditions provided to owned dogs over the past two decades in Costa Rica that offer benefits both to dog welfare and humans.

The 2020 survey found that almost three-quarters of households in Costa Rica currently own one or more dogs. As a result, more than 2 million dogs are predicted to be owned across the country. There was limited evidence that ownership patterns varied among provinces across the country but rather that ownership was better described by geography especially elevation, human population density, and dwelling type.

The factors that affect dog population numbers support an approach that would involve estimating dog population size across broad spatial scales. Dog ownership patterns also indicate where dog services might be best located to serve the companion animal industry. For example, we are aware of only a single animal shelter that, services a large portion of the San Jose central valley (approximately 1.5 million people). Our modeling could help to identify where shelters or veterinary clinics might best be located to serve community members. For instance, veterinary clinics may be located in areas with high dog ownership rates (e.g., areas dominated by single family homes with yards and high home ownership rates) whereas shelters may be located in areas prone to high relinquishment rates (e.g., areas with a high proportion of residents in rented homes). Furthermore, our estimates could be applied to understand how factors such as dog bites (which occurred in 16% of surveyed households in the past year) might be allocated among medical service facilities, especially if communities have more detailed spatial data of housing density, housing type, and ownership status that influences local dog abundance. Given the prevalence of dog ownership, considerations of the welfare of dogs and their owners should continue to garner future consideration by policy makers with respect to community planning, dog management, and veterinary service availability.

Very few owned dogs had free access to the streets at night or early in the morning and most owners reported that their dog was in the house during the night. Information such as this is important for community planning and monitoring the abundance and welfare of free-roaming dogs. First, our results indicate that management activities that occur during nighttime or early morning meant to target unowned free-roaming dogs are unlikely to encounter very many owned free-roaming dogs. For instance, in cases where dogs are trapped for sterilization activities (8), conducting these activities when the probability of capturing owned dogs is low is more likely to reduce issues with dog owners and maximize efforts toward the target population of unowned free-roaming dogs. Second, our results provide insights that are important for monitoring the free-roaming dog population from surveys that cannot differentiate the ownership status of dogs based on simple observations (22, 23). For example, surveys by observers to count free-roaming dogs in the early morning must make assumptions about the ownership of dogs based on features such as body coat or presence of collars (24, 25). Our data indicate that 33% of owned dogs do not wear collars and that only 1% of all free-roaming dogs roaming at night are actually owned. Therefore, only 0.33% of dogs observed during street surveys in Costa Rica at night should be wearing a collar. Given the lower probability of free access outside the home at night and morning with higher human population density, the proportion of owned dogs without collars will be even lower in the most populated urban areas. Third, the marked increase over 10 years from 27 to 66% of owned dogs being in the house overnight suggests that dog owner behavior has shifted toward reducing exposure and risk to owned dogs. Overall, this may indicate an improvement of welfare and living conditions of owned dogs in Costa Rica through a reduction in negative dog-dog or dog-wildlife interactions over the past two decades at the same time that dog ownership may have increased.

More than three quarters of owned dogs in Costa Rica visited a veterinarian for routine care over the past year. Compared to previous surveys in Costa Rica, these values are consistent over the past decade suggesting an upper boundary for dog owners obtaining regular veterinary care for their dog (In the USA, around two-thirds of owned dogs visit a veterinarian every year–AVMA 2017-2018). Given the proportion of dogs that had regular veterinary care, an almost equal proportion of dogs were sterilized and vaccinated. Interestingly, there were slight differences in sterilization rates between male and female dogs. Female dogs were more likely to be sterilized while male dogs were more likely to be vaccinated. Other studies in the USA also found female dogs have higher sterilization rates compared to males (26). These findings suggest some selectivity in acquiring veterinary services for owned dogs indicating that some dog owners will seek out veterinary care even if it is challenging (e.g., too expensive or considered too far to travel). At the same time, given that three quarters of dogs visited a veterinarian for routine care, there are likely some limiting factors that prevent other dog owners from accessing veterinary care. Identifying those limiting factors, and what actions could mitigate those factors, could contribute to a larger proportion of dog owners incorporating regular veterinary care into their dog ownership efforts. As animal advocacy grows in Costa Rica, anecdotal evidence indicates the number of requests for animal sterilization and vaccination services outside the San Jose valley have been increasing.

The above features generate confidence that Costa Rica could serve as an excellent case-study for a large-scale, integrated, cooperative, and scientifically robust humane management program for dogs. The production of a current dog population size estimate with some estimates of the scope of the roaming dog “problem” should lead to an informed estimate of the resources and the timeline required to achieve the desired outcomes. The baseline assessment provided in this study functions as a yardstick against which to measure progress as the dog management program moves forward. Both the government of Costa Rica and a well-established local NGO have demonstrated a commitment to a humane dog management program in the last two decades. This indicates excellent prospects for program sustainability.

It is not clear why the current survey has produced a much higher estimate of the owned dog population although the survey method may affect estimates of dog ownership and dog numbers. In the USA, Patronek and Rowan (27) noted that surveys using Random Digit Dial (RDD) methods produced estimates of dog ownership and total pet dog numbers that were about 20% lower than surveys using Household Panel methods (where households who have agreed to complete mail surveys are recruited to be on a large panel). Recently, UK Pet Food (28) in the UK reported pet dog and cat estimates from an online survey in the United Kingdom in 2021 that were 40–50% higher than the pet dog and cat estimates that had been developed from face-to-face surveys conducted from 2012 to 2020. In the USA, the American Pet Products Association has been conducting surveys of the US pet population every 2 years since 1988, In 2012, they switched from conducting surveys *via* Household Panel methods to using online survey technology. From 1988 to 2010, the APPA reported that 38–40% of US households had a pet but subsequent surveys reported an increasing percentage of households owning dogs (reaching 48% in the most recent survey). It is suggested that estimates of total pet populations are influenced by the survey methods used. It appears that online surveys produce higher pet population estimates compared to household panel or face-2-face survey methods. It is not obvious why this might be so but we suggest that consumers of pet survey data be aware that different survey methods might produce different pet population estimates.

The large changes in human dog care behavior in the 21^st^ Century indicated by the previous surveys (21) and our 2020 survey data are also supported by the growth in the membership of the veterinary college. According to reports from the Colegio de Veterinarios (the national oversight body for veterinary practice in Costa Rica). The number of new members joining the Colegio de Veterinarios annually increased from 10 in 1990 to 90 in 2010 (Karina Navarro, personal communication, 9 August 2017).

Our study, and the results drawn from a randomized questionnaire has some obvious limitations that warrant consideration. First, any survey is self-selecting as respondents cannot be forced to respond. We attempted to reduce the impact of this selection process by using a reputable survey company that maintains panels of respondents. These panels are assembled and assessed to ensure a represent sample can be obtained of the population of interest to reduce bias. We must also assume that all responses are true. Importantly, the questions asked were with regards to dog ownership and not with respect to dog management *per se*. Therefore, our findings should not be construed as indicating appropriate or effective management techniques or actions.

The changes in dog care and management are all in the direction of increasing control of, and care for, household dogs. The changes occurring in Costa Rica indicate that it is possible to evolve relatively quickly from a culture where dogs are mostly permitted to roam and receive relatively little veterinary care to a culture of “homed” pets. Similar changes in human-dog interaction and the control of pet dogs occurred in the USA in the last 25 years of the 20^th^ century when the number of unreturned (euthanized) unowned free-roaming dogs entering US shelters dropped from around 25% of the pet dog population to 5% (29).

## Data availability statement

The raw data supporting the conclusions of this article will be made available by the authors, without undue reservation.

## Author contributions

DF conducted the statistical analysis. All authors conceived the study, collected the data, and wrote and revised the manuscript. All authors contributed to the article and approved the submitted version.

## Funding

This study was funded by Laura J. Niles Foundation. The funders had no role in study design, data collection, analysis or interpretation of the data.

## Conflict of interest

DF was employed by Flockhart Consulting. AR was employed by Wellbeing International. JB was employed by Great Basin Bird Observatory.

## Publisher's note

All claims expressed in this article are solely those of the authors and do not necessarily represent those of their affiliated organizations, or those of the publisher, the editors and the reviewers. Any product that may be evaluated in this article, or claim that may be made by its manufacturer, is not guaranteed or endorsed by the publisher.
