# Supplementary material for: Owned dog population size and ownership patterns in Costa Rica
Source: Front Vet Sci. 2022 Dec 1;9:946603. doi: 10.3389/fvets.2022.946603 (PMC9751483; doi:10.3389/fvets.2022.946603)
Supplement: Supplementary file 1 [file Data_Sheet_1.pdf]

Owned dog population size and ownership patterns in Costa Rica

Supplementary Materials

D. T. Tyler Flockhart, Andrew N. Rowan[1], John D. Boone,

Corresponding author[1]: arowan@wellbeingintl.org

Appendix 1. Dog Population Model Methods & Results

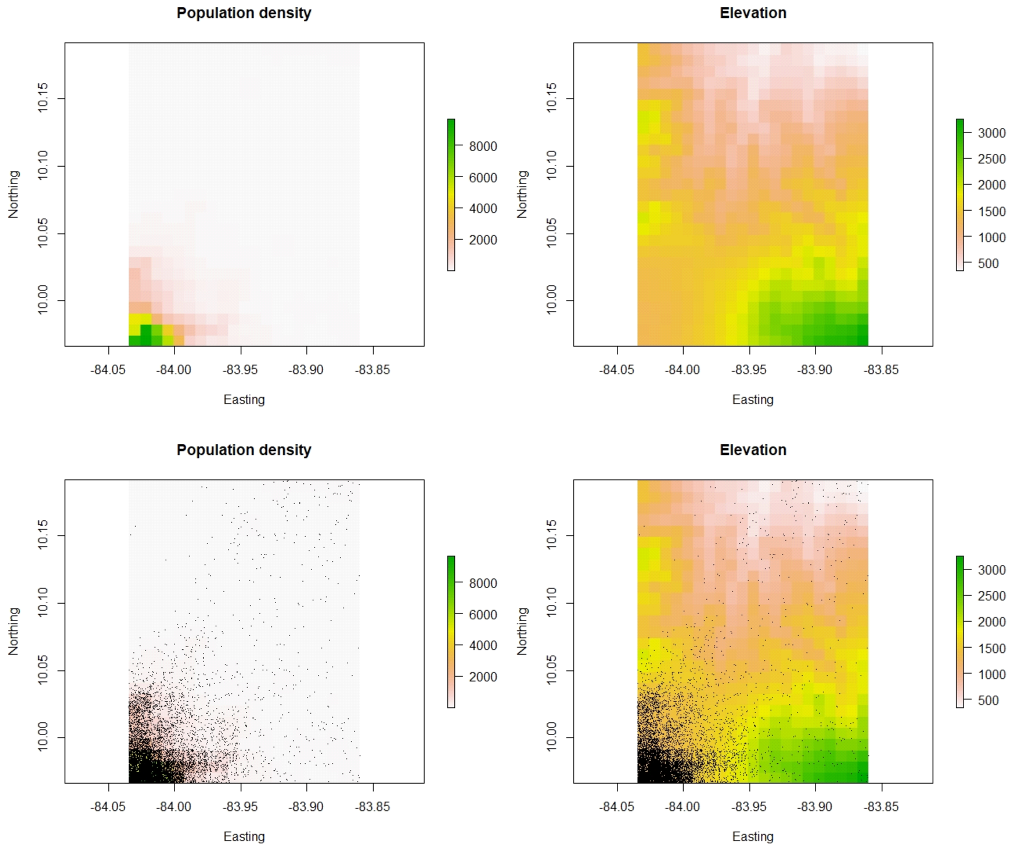

Figure S 1: The raw human population density per km^2 (top left) and elevation in meters (top right) spatial data sets for a given Canton and the overlay of 10,000 random locations weighted by the population density (bottom). The population density and elevation were extracted at each random point and used in bootstrap simulations.

Table S 1. Mean parameter estimates and 95% confidence intervals from the top model to explain the probability of dog ownership.

Parameter	Estimate	95% Confidence Interval
Intercept	2.469	0.945, 3.994
Dwelling type:		
Multi-unit	-1.823	-3.327, -0.318
Single family with yard	-0.954	-2.415, 0.506
Single family without yard	-2.246	-3.732, -0.760
Ownership:		
Own	0.642	0.165, 1.119
Rent	0.015	-0.472, 0.501
Population density	-0.000023	-0.000082, 0.000036
Elevation	-0.0099	-0.0021, 0.0001
Elevation2	0.000001	-9 x108, 0.0000013

Table S 2. Full candidate model list ranked using Akaike Information Criterion (AIC) to explain the probability of dog ownership of residents of Costa Rica. Variables considered include age (linear (Age) or quadratic (Age^2) form), land use, dwelling type, dwelling ownership status, population density, elevation (in linear or quadradic forms), household size, Province, and Canton. Indicated for each model are the number of model parameters (K), AIC value corrected for small sample size (AIC*c*), difference between AIC*c* for each model relative to the top model (ΔAIC*c*), model likelihood (L*i*), model weight (W*i*), log-likelihood (LL), and cumulative Akaike weight (∑Wt).

Model	K	AIC*c*	ΔAIC*c*	L*i*	W*i*	LL	∑Wt
Dwelling type + Ownership + Population density + $Elevation^2$	9	1367.1	0	1	0.34	-674.48	0.34
Dwelling type + Ownership + Population density + $Elevation^2$ + Land use	15	1367.6	0.5	0.78	0.27	-668.62	0.61
Dwelling type + Ownership + Population density + $Elevation^2$ + Household size	10	1368.32	1.22	0.54	0.19	-674.08	0.8
Dwelling type + Ownership + Population density + $Elevation^2$ + Household size + Land use	16	1368.84	1.74	0.42	0.14	-668.21	0.94
Dwelling type + Ownership	6	1370.62	3.52	0.17	0.06	-679.28	1
Dwelling type	4	1385.57	18.46	0	0	-688.77	1
Ownership	3	1432.68	65.58	0	0	-713.33	1
$Elevation^2$	3	1463.2	96.1	0	0	-728.59	1
Population density	2	1468.05	100.95	0	0	-732.02	1
Elevation	2	1468.32	101.22	0	0	-732.16	1
Province	7	1468.84	101.74	0	0	-727.38	1
Household size	2	1472.48	105.38	0	0	-734.23	1
Null	1	1473.05	105.95	0	0	-735.52	1
Age	2	1474.74	107.64	0	0	-735.37	1
Land use	7	1475.2	108.1	0	0	-730.56	1
Age^2	3	1475.78	108.68	0	0	-734.88	1
Cantons	78	1549.33	182.23	0	0	-691.81	1

Table S 3. Full candidate model list ranked using Akaike Information Criterion (AIC) to explain the number of dogs owned per household of residents of Costa Rica. Variables in the models include age in either a linear (Age) or quadratic (Age^2) form, land use, dwelling type, dwelling ownership status, population density, elevation (in linear or quadradic forms), household size, Province, and Canton. Indicated for each model are the number of model parameters (K), AIC value corrected for small sample size (AIC*c*), difference between AIC*c* for each model relative to the top model (ΔAIC*c*), model likelihood (L*i*), model weight (W*i*), log-likelihood (LL), and cumulative Akaike weight (∑Wt).

Model	K	AIC*c*	ΔAIC*c*	L*i*	W*i*	LL	∑Wt
Dwelling type * Ownership + Population density + $Elevation^2$+ Household size	16	4120.93	0	1	0.81	-2044.26	0.81
Dwelling type * Ownership + Population density + $Elevation^2$+ Household size	15	4125.32	4.38	0.11	0.09	-2047.48	0.9
Dwelling type + Ownership + Population density + $Elevation^2$+ Household size	10	4125.54	4.6	0.1	0.08	-2052.69	0.98
Dwelling type + Ownership + Population density + $Elevation^2$	9	4129.52	8.58	0.01	0.01	-2055.69	0.99
Dwelling type + Ownership + Population density + $Elevation^2$+ Household size + Land use	16	4130.59	9.65	0.01	0.01	-2049.09	1
Dwelling type + Ownership + Population density + $Elevation^2$ + Land use	15	4134.53	13.6	0	0	-2052.08	1
Dwelling type * Ownership	12	4139.88	18.94	0	0	-2057.82	1
Dwelling type + Ownership	6	4144.67	23.73	0	0	-2066.3	1
Dwelling type	4	4172.79	51.86	0	0	-2082.38	1
Ownership	3	4217.2	96.27	0	0	-2105.59	1
Population density	2	4253.55	132.62	0	0	-2124.77	1
$Elevation^2$	3	4259.95	139.02	0	0	-2126.97	1
Elevation	2	4265.06	144.13	0	0	-2130.53	1
Household size	2	4265.34	144.4	0	0	-2130.66	1

Cantons	78	4268.5	147.57	0	0	-2051.4	1
Null	1	4273.25	152.32	0	0	-2135.62	1
Province	7	4274.35	153.42	0	0	-2130.13	1
Age	2	4274.71	153.78	0	0	-2135.35	1
Age^2	3	4276.34	155.41	0	0	-2135.16	1
Land use	7	4277.29	156.36	0	0	-2131.6	1

Table S 4. Mean and 95% confidence interval of estimated number of owned dogs in each province in Costa Rica.

**Province**	**Mean**	**Lower 95% CI**	**Upper 95% CI**
Alajuela	453,508	352,788	576,956
Cartago	229,443	172,707	304,859
Guanacaste	174,608	138,037	219,764
Heredia	223,655	174,540	282,715
Limón	207,558	151,352	276,671
Puntarenas	229,137	178,752	289,094
San José	704,133	576,937	863,954

Table S 5. Mean and 95% confidence interval of estimated number of owned dogs in each canton in Costa Rica.

Canton	Mean	Lower 95% CI	Upper 95% CI
Abangares	9,559	4,107	15,198
Acosta	10,259	4,223	16,455
Aguirre	15,778	6,806	25,258
Alajuela	137,349	58,613	218,883
Alajuelita	33,799	14,405	53,997
Alfaro Ruíz	6,933	2,881	11,496
Alvarado	7,217	2,899	13,858
Aserrí	28,110	11,620	45,472
Atenas	13,836	5,906	21,982
Bagaces	10,786	4,585	16,914
Barva	19,664	8,229	32,928
Belén	11,134	4,751	17,752
Buenos Aires	23,309	9,780	39,013
Cañas	14,353	6,212	22,854
Carrillo	19,564	8,259	31,151
Cartago	65,611	28,222	107,861
Corredores	23,604	9,973	36,651
Coto Brus	20,755	8,883	33,798
Curridabat	34,089	14,467	53,833
Desamparados	98,816	41,117	163,270
Dota	4,189	1,607	8,343
El Guarco	19,367	8,242	31,734
Escazú	30,279	13,052	48,732
Esparza	17,161	7,177	27,025
Flores	10,192	4,301	16,045
Garabito	12,241	5,152	19,501
Goicoechea	55,887	23,536	92,884
Golfito	21,645	9,162	34,675
Grecia	41,388	17,611	65,879
Guácimo	24,825	10,500	39,545
Guatuso	8,813	3,810	14,056
Heredia	60,550	25,121	99,820
Hojancha	3,604	1,559	5,684
Jiménez	7,435	3,166	11,761
La Cruz	10,829	4,755	17,104
La Unión	46,085	19,276	71,926
León Cortés Castro	6,567	2,786	10,654
Liberia	32,175	13,780	51,528

Limón	45,238	18,816	72,736
Los Chiles	13,821	5,811	22,160
Matina	20,643	8,767	32,600
Montes de Oca	31,211	13,285	49,724
Montes de Oro	6,900	2,882	10,910
Mora	14,241	5,938	22,530
Moravia	27,185	11,325	44,447
Nandayure	5,636	2,422	8,987
Naranjo	21,447	9,004	33,497
Nicoya	24,981	10,029	41,343
Oreamuno	20,699	8,500	36,476
Orotina	11,078	4,795	17,443
Osa	14,977	6,244	23,133
Palmares	17,900	7,706	28,217
Paraíso	26,626	11,152	44,431
Parrita	9,539	4,036	14,884
Pérez Zeledón	67,175	28,139	114,533
Poás	14,541	6,220	23,665
Pococí	68,347	28,940	108,903
Puntarenas	63,228	26,961	99,650
Puriscal	17,903	7,576	28,341
San Carlos	87,899	37,650	142,311
San Isidro	10,706	4,534	17,256
San José	139,505	59,524	217,413
San Mateo	3,311	1,382	5,223
San Pablo	13,190	5,587	20,768
San Rafael	23,836	9,784	38,847
San Ramón	42,456	17,601	68,979
Santa Ana	26,929	11,400	42,454
Santa Bárbara	18,297	7,689	29,616
Santa Cruz	32,492	13,597	51,276
Santo Domingo	20,517	8,617	32,304
Sarapiquí	35,570	15,007	56,307
Siquirres	30,015	12,391	48,037
Talamanca	18,490	7,629	31,772
Tarrazú	8,774	3,677	14,151
Tibás	35,662	15,330	56,925
Tilarán	10,624	4,554	16,587
Turrialba	36,402	15,156	62,669
Turrubares	3,307	1,419	5,289
Upala	22,833	9,717	36,161

Valverde Vega	9,905	4,137	15,742
Vázquez de Coronado	30,237	12,783	51,298

Table S *6*. Full candidate model list ranked using Akaike Information Criterion (AIC) to explain where dogs sleep based on a sample of dog owners in Costa Rica. Variables in the models include age in either a linear (Age) or quadratic (Age^2) form, land use, dwelling type, dwelling ownership status, population density, elevation (in linear or quadradic forms), household size, Province, and Canton. Indicated for each model are the number of model parameters (K), AIC value corrected for small sample size (AIC*c*), difference between AIC*c* for each model relative to the top model (ΔAIC*c*), model likelihood (L*i*), model weight (W*i*), log-likelihood (LL), and cumulative Akaike weight (∑Wt).

Model	K	AIC*c*	ΔAIC*c*	L*i*	W*i*	LL	∑Wt
$Elevation^2$ + Population density + Dwelling type	21	2729.94	0	1	0.59	-1343.7	0.59
$Elevation^2$ + Dwelling type	18	2731.59	1.65	0.44	0.26	-1347.6	0.85
$Elevation^2$ + Population density + Dwelling type + Province	39	2733.34	3.4	0.18	0.11	-1326.74	0.96
$Elevation^2$ + Dwelling type + Province	36	2735.21	5.26	0.07	0.04	-1330.81	1
$Elevation^2$ + Population density	12	2759.84	29.9	0	0	-1367.83	1
$Elevation^2$ + Population density + Province	30	2763.76	33.82	0	0	-1351.33	1
$Elevation^2$	9	2765.31	35.37	0	0	-1373.6	1
$Elevation^2$ + Province	27	2768.87	38.93	0	0	-1356.99	1
Elevation	6	2772.85	42.91	0	0	-1380.4	1
Population density	6	2776.55	46.61	0	0	-1382.25	1
Dwelling type	12	2778.17	48.23	0	0	-1376.99	1
Province	21	2779.04	49.1	0	0	-1368.25	1
Ownership	9	2791.54	61.6	0	0	-1386.72	1
Age	6	2792.8	62.86	0	0	-1390.37	1
Number of owned dogs	6	2796.03	66.09	0	0	-1391.99	1
Age^2	9	2796.16	66.22	0	0	-1389.03	1
Land use	18	2809.82	79.88	0	0	-1386.71	1
Household size	6	2810.42	80.48	0	0	-1399.19	1
Null	3	2815.48	85.54	0	0	-1404.73	1

Cantons	234	2953.78	223.84	0	0	-1205.78	1

Table S 7. Mean parameter estimates and 95% confidence intervals from the top model to explain where dogs' sleep.

	Confined in yard		On street		Unconfined in yard	
	Estimate	95% CI	Estimate	95% CI	Estimate	95% CI
Intercept	-2.026	-2.026, -2.026	-6.404	-6.404, -6.404	0.989	0.989, 0.989
Elevation	-0.002	-0.002, -0.002	-0.001	-0.001, -0.001	-0.001	-0.002, -0.001
$Elevation^2$	5.091e-07	2.490e-07, 7.693e-07	4.420e-07	-1.709e-07, 1.055e-06	5.173e-07	1.724e-07, 8.622e-07
Population density	-7.9374e-05	-0.0002, 8.7223e-06	-0.0002	-0.0005, 2.4180e-05	5.410e-07	-0.0001, -4.8256e-06
Dwelling type:						
Multi-unit building	0.424	0.424, 0.424	4.504	4.504, 4.504	-1.565	-1.565, -1.565
Single family with yard	0.747	0.747, 0.747	2.919	2.919, 2.919	-1.146	-1.146, -1.146
Single family without yard	-0.0454	-0.0454, -0.0454	3.147	3.147, 3.147	-2.191	-2.191, -2.191

Table S 8. Full candidate model list ranked using Akaike Information Criterion (AIC) to explain where a dog is at 8am sleep based on a sample of dog owners in Costa Rica. Variables in the models include age in either a linear (Age) or quadratic (Age^2) form, land use, dwelling type, dwelling ownership status, population density, elevation (in linear or quadradic forms), household size, Province, and Canton. Indicated for each model are the number of model parameters (K), AIC value corrected for small sample size (AIC*c*), difference between AIC*c* for each model relative to the top model (ΔAIC*c*), model likelihood (L*i*), model weight (W*i*), log-likelihood (LL), and cumulative Akaike weight (∑Wt).

Model	K	AIC*c*	ΔAIC*c*	L*i*	W*i*	LL	∑Wt
$Elevation^2$ + Population density + Dwelling type	21	2868	0	1	0.98	-1412.72	0.98
$Elevation^2$ + Population density + Dwelling type + Province	39	2876.35	8.35	0.02	0.02	-1398.24	1
$Elevation^2$ + Dwelling type	18	2892.28	24.28	0	0	-1427.94	1
$Elevation^2$ + Dwelling type + Province	36	2896.07	28.08	0	0	-1411.24	1
Dwelling type	12	2923.83	55.83	0	0	-1449.82	1
$Elevation^2$ + Population density	12	2924.29	56.29	0	0	-1450.05	1
$Elevation^2$ + Population density + Province	30	2929.11	61.11	0	0	-1434	1
Population density	6	2929.19	61.2	0	0	-1458.57	1
$Elevation^2$ + Province	27	2954.85	86.85	0	0	-1449.98	1
$Elevation^2$	9	2956.33	88.33	0	0	-1469.11	1
Province	21	2966.79	98.8	0	0	-1462.12	1
Age	6	2968.62	100.62	0	0	-1478.28	1
Elevation	6	2970.02	102.02	0	0	-1478.98	1
Age^2	9	2971.48	103.49	0	0	-1476.69	1
Number of owned dogs	6	2976.25	108.26	0	0	-1482.1	1
Land use	18	2989.33	121.33	0	0	-1476.46	1
Household size	6	2989.52	121.53	0	0	-1488.74	1
Null	3	2992.09	124.09	0	0	-1493.04	1
Ownership	9	2994.67	126.68	0	0	-1488.28	1

Cantons	234	3089.9	221.91	0	0	-1273.85	1

Table S 9. Mean parameter estimates and 95% confidence intervals from the top model to explain where a dog is at 8am.

	Confined in yard		On street		Unconfined in yard	
	Estimate	95% CI	Estimate	95% CI	Estimate	95% CI
Intercept	-2.389	-2.389, -2.389	-12.262	-12.262, -12.262	0.640	0.640, 0.640
Elevation	0.00196	0.00193, 0.00199	-0.00147	-0.00149, -0.00144	-0.00058	-0.00108, -6.85103e-05
Elevation2	-1.737e-06	-2.166e-06, -1.309e-06	6.232e-07	2.147e-07, 1.032e-06	2.912e-07	-4.860e-08, 6.311e-07
Population density	-0.00015	-2.8e-04, -1.874e-05	-1.467e-04	-3.029e-04, 9.533e-06	-1.209e-04	-1.659 e-04, -7.588e-05
Dwelling type:						
Multi-unit building	-1.512	-1.512, -1.512	10.693	10.693, 10.693	-0.845	-0.845, -0.845
Single family with yard	0.215	0.215, 0.215	9.701	9.701, 9.701	-0.094	-0.094, -0.094
Single family without yard	-1.732	-1.732, -1.732	8.739	8.739, 8.739	-1.667	-1.667, -1.667

Appendix 2. Questionnaire used for Household Survey.

Location

1. Household Location
    a. Map (place pin for home location)
2. What is your home address?
3. In which province is your home?
    a. Alajuela
    b. Cartago
    c. Guanacaste
    d. Heredia
    e. Limon
    f. Puntarenas
    g. San Jose
4. In which Canton and District (if known) is your home?

Household or Owned Dogs

5. How many dogs does your household own?

Please complete the following for up to three dogs (choose the three most recently acquired dogs)

6. Gender (Dog 1, Dog 2, Dog 3)
    a. Male
    b. Female
7. Sterilized (Dog 1, Dog 2, Dog 3)
    a. Yes
    b. No
8. Vaccinated in last 12 months (Dog 1, Dog 2, Dog 3)
    a. Yes
    b. No
9. Visited veterinarian in previous 12 months (Dog 1, Dog 2, Dog 3)
    a. For routine care
        i. Yes
        ii. No
    b. For emergency treatment
        i. Yes
        ii. No
10. Routinely wears a collar (Dog 1, Dog 2, Dog 3)
    a. Yes
    b. No
11. Where do your dogs sleep at night? (Dog 1, Dog 2, Dog 3)

    a. In house
    b. Confined/tethered in yard
    c. Unconfined in yard
    d. On the street
    e. Unknown

12. Where are your dogs normally between 6 and 9 am? (Dog 1, Dog 2, Dog 3)
    a. In house
    b. Confined/tethered in yard
    c. Unconfined in yard
    d. On the street
    e. Unknown

Street or Unowned Dogs

13. How many dogs in your neighborhood that are not owned by your household are given food once a week or more often?
14. Approximately how many dogs are seen on the streets around your home?
    a. None
    b. 1 to 2
    c. 3 to 5
    d. 6 or more
15. What should be done about street dogs?
    a. Nothing
    b. Catch vaccinate & return to street
    c. Catch vaccinate sterilize & return to street
    d. Catch and move elsewhere
    e. Catch and euthanize
    f. Do not know

Dog Bites

16. How many persons (adults and children) in your household have been bitten by a dog (whether owned or unowned) in the last 12 months?
        i. None
    b. One
    c. Two
    d. Three or more

Household Information:

17. How many adults (18 and over) live in the household?
18. How many adults in the household were born in Costa Rica?
    a. All
    b. Some

    c. None
    d. Unknown
19. How many children (under 18) live in the household?
20. What type of household do you live in?
    a. Single family with yard
    b. Single family without yard
    c. Multi-unit building
    d. Farm
    e. Other
21. Do you own or rent your current home?
    a. Own
    b. Rent
    c. Other
    d. Unknown
